# Integrated Electromechanical Transduction Schemes for Polymer MEMS Sensors

**DOI:** 10.3390/mi9050197

**Published:** 2018-04-24

**Authors:** Damien Thuau, Pierre-Henri Ducrot, Philippe Poulin, Isabelle Dufour, Cédric Ayela

**Affiliations:** 1 Laboratoire IMS, University Bordeaux, UMR 5218, ENSCBP, 16 Avenue Pey Berland, 33607 Pessac Cedex, France; pierre-henri.ducrot@outlook.fr (P.-H.D.); isabelle.dufour@ims-bordeaux.fr (I.D.); cedric.ayela@ims-bordeaux.fr (C.A.); 2 Centre de Recherche Paul Pascal, University Bordeaux, Avenue Schweitzer, 33600 Pessac, France; poulin@crpp-bordeaux.cnrs.fr

**Keywords:** electromechanical transduction, polymer MEMS, piezoresistivity, piezoelectricity, Piezo-organic field effect transistor

## Abstract

Polymer Micro ElectroMechanical Systems (MEMS) have the potential to constitute a powerful alternative to silicon-based MEMS devices for sensing applications. Although the use of commercial photoresists as structural material in polymer MEMS has been widely reported, the integration of functional polymer materials as electromechanical transducers has not yet received the same amount of interest. In this context, we report on the design and fabrication of different electromechanical schemes based on polymeric materials ensuring different transduction functions. Piezoresistive transduction made of carbon nanotube-based nanocomposites with a gauge factor of 200 was embedded within U-shaped polymeric cantilevers operating either in static or dynamic modes. Flexible resonators with integrated piezoelectric transduction were also realized and used as efficient viscosity sensors. Finally, piezoelectric-based organic field effect transistor (OFET) electromechanical transduction exhibiting a record sensitivity of over 600 was integrated into polymer cantilevers and used as highly sensitive strain and humidity sensors. Such advances in integrated electromechanical transduction schemes should favor the development of novel all-polymer MEMS devices for flexible and wearable applications in the future.

## 1. Introduction

The last 30 years have seen the advent of MicroElectroMechanical Systems (MEMS) for a wide range of applications, including digital projectors, inertial (automotive, joysticks, phones) and chemical sensors. Most often, the substrate used as a base layer is silicon, characterized by intrinsic mechanical and electrical properties that are not necessarily the most adequate for a given application. For example, when resonant silicon cantilevers are used for sensing in liquid media, damping makes motion measurements hard to achieve, while in the static mode, silicon-based (Si) cantilever sensors are restrictively stiff, as opposed to polymer MEMS. Thanks to their tailor-made synthesis or formulation enabling to finely tune their properties, polymer materials integrated into MEMS have the potential to constitute a powerful alternative to Si based-MEMS devices, just like organic light-emitting diodes (OLEDs) are taking a major share of the global market of the smartphone and display industries [1]. To develop original polymer MEMS devices, their design and fabrication have to be rethought with respect to Si MEMS and associated micro- and nanomachining methods [2]. In particular, the integration of the transduction is crucial to taking advantage of the high mechanical deformability of polymer materials while maintaining the low volume of the devices offered by miniaturization [3]. Recent advances in materials science and mechanical engineering enable the use of polymers as functional materials for efficient electromechanical transduction and for their integration into polymeric MEMS [4]. For instance, the development of the next generation of M/NEMS sensors requires cutting-edge strategies in their design and fabrication, both at the level of integration and material development. The development of polymer M/NEMS with associated printing fabrication methods meets the requirements for rapid prototyping, enabling mass production, at reduced cost. Pioneering work was conducted by Boisen’s group and Ramgopal Rao’s group on the fabrication by photolithography and application of microcantilevers made of SU-8, an epoxy photoresist widely used in microfluidics [5]. Since then, different approaches have been specifically developed for the fabrication of free-standing polymer MEMS. These include lamination [6], molding [7], ink-jet printing [8], nano-imprint lithography [9] and shadow-masking [10]. However, the wafer-level fabrication of free-standing organic cantilevers remains challenging. Approaches are based on UV barriers [11], light absorption properties of photoresists [12] and wafer-bonding processes [13]. Recently, two-photon stereolithography (TPS) enabled single-step fabrication of complex organic MEMS, such as microfluidic resonators [14]. One example can be found in the literature where SU-8 was used as structural material [15].

Integrated actuation and read-out schemes for MEMS devices are of particular importance for maintaining the typically small size of such microsystems, enabling their portability. When MEMS are used as resonators, actuation to the resonance and motion read-out using the same scheme is the best configuration in terms of simplicity and size. The most popular schemes are electrostatic actuation and capacitive read-out, piezoelectricity, and electromagnetism [16]. However, in the field of organic MEMS, split actuation and read-out are still more common. For several years, piezoresistive MEMS have generally been made of metal or semiconductor materials (i.e., doped Silicon) exploiting respectively the change of resistance due to geometrical variations of the free-standing devices or modification of the width of the band-gap and consequently the mobility of the charge carriers. Nevertheless, the poor mechanical elasticity of inorganic materials limits their sensitivity. To counter this drawback, a recent trend consists of exploring polymeric materials like conducting polymers such as PEDOT/PSS [17] or carbon-related materials (e.g., carbon black, carbon nanotube, graphene) often used as fillers in a nanocomposite formed with additional polymer (polystyrenes, SU-8, polyimide, PDMS) [18,19,20,21]. The first example of concrete application of SU-8 micro-cantilevers in the framework of gas sensing consisted of piezoresistive composite based (mixture of SU-8 and black carbon) cantilevers used for detection of TNT in static mode [22]. These results showed promising polymeric gas sensors, with an achieved limit of detection of 14ppb, a value close to traces. Later on, they reported a polymer MEMS accelerometer based on the same piezoresistive composite. The fabricated devices exhibited a resonant frequency of 10.8 kHz and a response sensitivity of 280 nm·g^−1^ at resonance [23]. Transparent MEMS pressure sensors made of piezoresistive carbon nanotube film embedded into PDMS membranes were also reported, with a gauge factor of 10–12 [24]. Among carbon-related materials, carbon nanotubes (CNTs) are seen as the most prominent fillers in polymer composites. Their high aspect ratio allows CNTs to create, at low loading, a more efficient conductive network within the insulting polymer matrix compared to other common fillers. Although the conductive network may be brittle for large deformations, this turns out to be an advantage for low strain sensing application where the network configuration is therefore very sensitive to mechanical disturbances, resulting in large changes of electrical resistance at CNT concentrations near the percolation region, leading to a high gauge factor [25,26,27,28,29]. Nevertheless, piezoresistive materials are defined as passive transduction, meaning that it led to a variation of an impedance without active electrical production (charge, current of voltage). Moreover, passive transduction requires a Wheatstone bridge electronics configuration for electrical signal read-out. On the contrary, one of the main advantages of using piezoelectric material lies in the reversible piezoelectric effect which allows both integrated actuation and read-out for dynamic mode measurement. In fact, piezoelectric MEMS are another common type of microsystem mainly used in automotive and smart phones applications [30]. These microsystems are often made of inorganic piezoelectric materials, the most common being lead zirconate titanate (PZT). However, PZT requires high-cost manufacturing capabilities for fabrication. Furthermore, lead-containing materials such as PZT present a certain toxicity. Consequently, recent research works have been oriented towards lead-free piezoelectric materials such as zinc oxide (ZnO) [31] or BaTiO_3_ [32], often used as nanowires or nanoparticles in a composite approach showing excellent performances notably for energy harvesting applications [33]. Nevertheless, preparation and polarization of these materials require high-temperature processing. Consequently, piezoelectric polymeric materials such as poly(vinylidenedifluoride), P(VDF), and more particularly its copolymer poly(vinylidenedifluoride-co-trifluoroethylene), P(VDF-TrFE), have attracting growing interest. In fact, the introduction of trifluoroethylene (TrFE) units to the P(VDF) leads to a direct crystallization into a crystal structure similar to that of the β-phase of PVDF, which consequently yields a material with a high piezoelectric effect. Although their electromechanical couplings are significantly lower than their inorganic counterpart, piezoelectric polymers are less expensive in terms of material cost and processing facilities by means of printing technologies [34,35]. Piezoelectric polymer-based MEMS also find applications in soft, flexible/stretchable formats, with unique opportunities for use in biological applications as well as mechanical energy harvesting [36,37]. Note that despite their numerous advantages, piezoelectric polymer transducers exhibit a limited sensitivity in contrast to polymer piezoresistors. Other actuation schemes include electrostatic actuation, made possible if the cantilever is (in part) made of conductive polymer, suspended above a metallic electrode [38]. Also, Schmid’s group introduced a scheme based on a polarization force induced in a dielectric polymer [39]. This uses the spontaneous polarization of dielectric polymers under high electric field to create an actuation force. Electromagnetic actuation has also been investigated using a conducting path patterned on top of an organic cantilever, allowing Lorentz force actuation when subjected to a constant magnetic field [40]. This scheme is clearly efficient, but read-out is a challenge, although it is possible by generation of inductive current due to the motion of the MEMS.

Since sensitivity is a pressing concern for MEMS sensors, these prevailing integrated electromechanical transduction mechanisms are today in competition with transistive transduction schemes. Transistive electromechanical transduction based on field effect transistor (FET) offers a number of advantages over traditional piezoresistive or capacitive MEMS, because of its high sensitivity, uncomplicated current measurement and compatibility with integrated circuits [41,42,43,44]. Pioneer inorganic transistive transduction has also been adapted to an organic approach by the integration of an organic field effect transistor (OFET) into a SU-8 polymeric cantilever [45]. Later on, with the aim to further enhancing the sensitivity of the MEMS sensors, piezotransistive electromechanical transduction read-out was reported, where piezoelectric gated OFET were able to directly utilize the charge density variation caused by the deflection-induced strain to induce amplified modulation of the OFET drain current [46,47].

In this paper, we report our recent advances in electromechanical transduction mechanisms for polymer MEMS sensors. All of them were made from organic materials, and their associated fabrication processes were compatible with polymeric microsystems, which greatly facilitates their integration as electromechanical transducers in multilayered structures. The presented polymeric integrated electromechanical transduction schemes offer features (high sensitivity, deformability, and biocompatibility) that significantly broaden the possibilities of these emerging MEMS.

## 2. Materials and Methods

Piezoresistive CNT/SU-8 nanocomposite cantilever: CNTs used in this work were supplied by using Graphistrength Epoxy Master batch pellets from Arkema (Colombes, France). The pellets consist in 25 wt % of MWNTs made via catalytic chemical vapor deposition (CCVD) dispersed in an epoxy-type matrix. The nanocomposites were prepared by mixing the SU-8 epoxy photoresist with the pellets using a high shear mixer, Silverson L4RT (Silverson SA, Evry, France) at 5000 rpm for 60 min in an ice bath. Then, piezoresistive polymeric MEMS were fabricated via two distinct approaches. The first one consists of an optimized photolithography process according to the CNT concentration integrated within the SU-8 epoxy resin, thus altering the UV crosslinking of the composite as described in [28]. The other fabrication approach is based on a low-environmental-impact approach in which microsystems were patterned by xurography [48]. Here, the piezoresistive solution was spin-coated on a sheet of 100 µm thick of Polyethylene terephthalate (PET) and soft-baked at 95 °C for 2 min. Then, the thin film was cross-linked by exposure to UV light before a post-exposure bake step at 65 °C for 1 min and 95 °C for 3 min followed by final hard bake of 150 °C for 15 min. Afterwards, the resonators were patterned using a vinyl cutting machine (Graphtec Craft ROBO Pro CE 5000-4, (PromatTex, Neuilly-sur-Marne, France).

P(VDF-TrFE) Piezoelectric polymer cantilever: 30 nm of aluminum was evaporated through a PET shadow masks to pattern the bottom electrode on a 50 μm thick Polyethylene naphthalate (PEN) film, used as a substrate. Then, a 4 µm thick PVDF-TrFE layer was deposited by solution process using a solution of 20 wt % of PVDF-TrFE (75–25% in mole), from Piezotech, dissolved in 2-butanone. Two annealing steps were then performed: first, at 50 °C for 10 min to evaporate the solvent, followed by a 140 °C period of varying durations. The top aluminum electrode was then evaporated under the same conditions as the bottom one through a PET shadow mask. To finish the process, the shape of the cantilever was obtained simply by cutting the material by xurography before gluing the resulting device onto a glass blade with double-sided adhesive tape, leaving the cantilever free-standing.

*Piezoelectric gate dielectric-based OFET cantilever:* The piezoelectric OFET integrated into a micro-cantilever made of flexible PEN has a bottom-gate top-contact structure, which consists of an Aluminum (Al) gate electrode, P(VDF-TrFE) and poly(1-vinyl-1,2,4-triazole) (PVT) gate dielectric layers, an organic semiconductor (OSC) and a Gold (Au) source-drain (S/D) electrodes. The entire stacking was encapsulated with a thin layer of tetratetracontane (TTC, C_44_H_90_). OFET-embedded MEMS were fabricated by combining classical deposition techniques with xurography. First, Al was evaporated through a shadow mask to pattern gate electrodes. P(VDF-TrFE) piezoelectric copolymer employed as gate dielectric combined with PVT used as dielectric passivation layer was deposited by spin coating. Afterwards, two well-known p-type organic semiconductors, pentacene and dinaphtho [2,3-b:2,3-f] thieno [3,2–b] thiophene (DNTT), an air-stable organic semiconductor, were thermally evaporated under secondary vacuum with a thickness of 30 nm. 60 nm thick Au source and drain contacts were thermally evaporated through shadow masks. The last step consisted in the evaporation of TTC, a long alkane chain molecule generally used as gate dielectric and employed in this work as a thin encapsulation layer for air stability measurements.

Strain sensor characterization: The piezoresistive sensitivity of the fabricated devices was electromechanically characterized. The protocol consisted of bending either the piezoresistive or piezotranstive cantilever beam by applying a force at the cantilever’s tip from a MiBot probe (Imina Technologies SA, Lausanne, Switzerland), meanwhile measuring the impedance evolution of the piezoresistor or drain current variation respectively to evaluate the sensor’s sensitivity. Optical profilometry images of the cantilever at rest and bent under an applied force corresponding to a tip-end cantilever beam deflection were obtained using a Veeco NT 9080 (VEECO, Plainview, USA).

Humidity sensor characterization: Different levels of relative humidity between 20 and 80% were generated from a Eurotherm 2604 controlled environmental chamber from Surface Measurement Systems^®^ while the temperature was kept constant at 20 °C during the experiment. The drain current variation of the embedded OFET was measured using a semiconductor analyzer Keithley 2400.

## 3. Results and Discussion

### 3.1. Piezoresistive Transduction

#### 3.1.1. Static Mode

**Figure 1:**
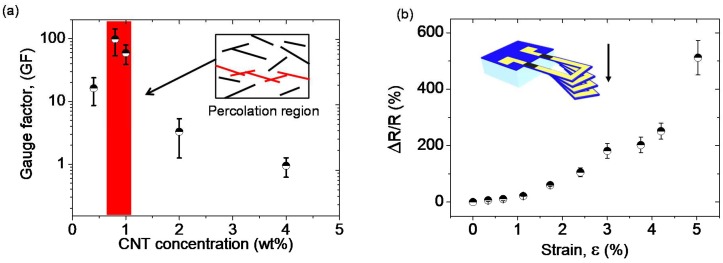
(**a**) Gauge factors of CNT/SU-8 composites as a function of CNT concentrations at 3.0% of applied strain, highlighting the highest sensitivities obtained at the percolation region; (**b**) Relative change of resistance of piezoresistive MEMS as a function of applied strain for a CNT concentration of 2 wt %.

The sensitivity of a piezoresistive material is defined by its gauge factor (*GF*) and can be expressed as:

$$GF=\frac{\Delta R/R}{\Delta \varepsilon }$$

where *R* is the initial resistance of the thin film at rest, Δ*R* the change of resistance under strain and Δ*ε* the strain variation. In order to investigate the sensitivity of the conformable CNT/SU-8 strain sensor, the relative change of resistance as a function of applied strain for thin films containing various CNT concentrations was measured. The resistance of all the samples increases as the applied strain increases. This arises from the alteration of the network of carbon nanotubes under mechanical deformation resulting in an increase in the resistivity. This change of resistivity is particularly associated with the modification of contact arrangements and the tunneling distance between carbon nanotubes. Figure 1a shows the gauge factor defined as the sensitivity to strain of different composites. The highest sensitivity (*GF*) was obtained for samples containing a CNT concentration just above the percolation threshold. This is due to the fact that the piezoresistive behavior is essentially governed by changes of the nanotube connectivity resulting in large variation of the tunneling resistance, *R_t_*. More specifically, 0.8 wt % CNT/SU-8 thin films have shown gauge factor of approximately 100 for a strain level of 3.0%. As the CNT concentration moves away from the percolation threshold the gauge factors have been found to decrease drastically.

Such a piezoresistive CNT/SU-8 material was subsequently integrated as transducer in bimorph polymeric SU-8 cantilevers. The piezoresistive composite was patterned by photolithography at the anchor of the beam while the ending part of the U-shaped beam was patterned with gold to minimize the electrical noise level in the piezoresistor. Since gold has a low resistivity, the initial resistance of the piezoresistor, *R*, is reduced, and the relative resistance change, Δ*R*/*R*, optimized. Nevertheless, a divergence of the piezoresistivity between the macroscale thin films and the integrated piezoresistive strain gauges of the MEMS sensors was observed. Although higher sensitivity was obtained for thin films containing 0.8 wt % CNT, the most sensitive piezoresistive MEMS were achieved using 2 wt % CNT. Additionally, the small dimensions of integrated piezoresistors in MEMS devices with higher intrinsic resistance made the measurements of 0.4 and 0.8 wt % CNT/SU-8 thin films difficult (resistance in the range of few hundreds MΩ to GΩ). Concretely, the integrated piezoresistive MEMS exhibited a non-linear electromechanical response, as shown in Figure 1b. At low strain level (<1.5%), gauge factors of 10 were measured. At larger strain level, gauge factors increased significantly. For instance, a gauge factor of 200 was calculated at *ε* = 4%. These results demonstrate the promise of nanocomposite approaches in tailoring and enhancing the sensitivity of piezoresistive electromechanical transduction read-out.

#### 3.1.2. Dynamic Mode

**Figure 2:**
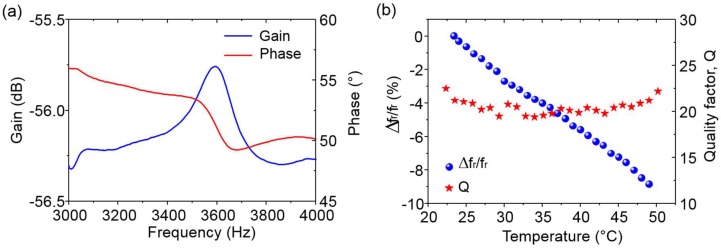
Electrical measurements of (**a**) Amplitude and phase of the resonant frequency of the first out-of-plane flexural mode measured by piezoresistive detection; and (**b**) Relative shift of resonant frequency and quality factor as a function of temperature.

Bimorph polymeric cantilevers were fabricated on PEN covered with a CNT/SU-8 piezoresitive layer. These devices were successfully driven into resonance by an external piezoelectric actuation while dynamic piezoresistive transduction was measured and compared to optical detection where the vibration amplitudes at resonance were detected with a laser vibrometer Polytec MSA 500 (Polytec SAS, Chatillon, France). The piezoresistive measurements were recorded with a network analyzer Agilent E5061B, (Agilent Technologies, Santa Clara, CA, USA) where the MEMS were tested in a half Wheatstone bridge configuration without using any amplification. All measurements were performed in air at atmospheric pressure. The resonant frequencies of the first out-of-plane flexural mode was measured at 3602 and 3597 Hz for piezoresistive and optical detection, respectively, thus showing excellent dynamic electromechanical transduction of the piezoresistive nanocomposite (Figure 2a). Quality factor (Q), defined as the ratio of the resonant frequency to the bandwidth associated with a 3 dB magnitude drop, was calculated to be 23 with optical read-out. In the case of piezoresistive detection, Q could not be determined classically due to the low amplitude of the resonant peak mainly due to spurious contributions. For systems with low Q factor, the measurement precision suffers from various unwanted spectral components induced by parasitic effects of the resonator and measurement errors. In order to extract the quality factor, we used an analytical model developed by Niedermayer et al. [49] to decompose the measured spectrum and determine the parameters of the second-order resonance and the background spectrum. An extracted Q value of 21 was obtained. The homogeneity of the resonant frequency and quality factor values obtained by optical and piezoresistive read-outs demonstrated the good dynamic operation of the fabricated polymer piezoresistive MEMS resonators.

These piezoresitive MEMS resonators were used as temperature sensors with a sensitivity of −0.33% per °C in the range of temperature 20–50 °C and a limit of detection of 0.112 °C (Figure 2b), which is comparable to the best commercial Pt100 Class AA temperature sensors. The decrease of resonant frequency with temperature is due to the softening of the materials, the resonant frequency of a resonator being directly proportional to the square root of the Young’s modulus of its materials. As expected, as temperature increases, the resonant frequency of the device decreases. We showed in a previous work [29] that monitoring the change of resonant frequency and quality factor of such resonator over a large range of temperature could lead to an excellent thermomechanical platform to characterize materials properties in a similar manner to dynamic mechanical analysis (DMA) measurements. Considering the cost, simplicity of fabrication, versatility and performance, the proposed MEMS offers exciting new possibilities across a wide spectrum of applications, including microsensors and micro-actuators.

### 3.2. Piezoelectric Polymer Transducer

To enable the simultaneous actuation and read-out of polymer MEMS resonators, P(VDF-TrFE) piezoelectric resonators were fabricated and their resonant frequency measured from admittance spectra. A dedicated electronic interface that operated as a Vectorial Network Analyzer (VNA) was developed and employed for both actuation and read-out of the resonant spectrum. Concretely, two cantilevers (a free one and an unreleased one) constitute a capacitive half-bridge. The signal from the common electrode is sent to a charge amplifier and then goes to an IQ demodulator that gives the amplitude and phase spectra. Such conditioning electronic card enables good compensation of the parasitic capacitance due to the dielectric layer (PVDF-TrFE) lying between the two conductors (the electrodes). To evaluate the ability of the dynamic piezoelectric transduction of the polymeric MEMS, we tested the fabricated devices as viscosity sensors in liquid media. Theoretically, it is possible to determine both the viscosity and the mass density of a fluid by measuring both the resonance frequency, *f_r_*, and the quality factor, *Q*, of the MEMS resonators. In this case, six parameters (*m*_1_, *m*_2_, *m*_3_, *c_1_, c*_2_ and *c*_3_) must be calculated [50]:

$$\frac{1}{{\left(2\pi f_{r}\right)}^{2}}=m_{1}+m_{2}\rho +m_{3}\sqrt{\frac{\eta \rho }{2\pi f_{r}}}$$

$$\frac{1}{Q}=2\pi f_{r}\left(c_{1}+c_{2}\eta +c_{3}\sqrt{2\pi f_{r}\eta \rho }\right)$$

with *η* the viscosity and *ρ* the mass density of the fluid. It means that a calibration with three liquids whose mass densities and viscosities are known is necessary to determine these coefficients. Then, the viscosity and the mass density can be analytically and independently determined for unknown liquids [51]:

$$\rho =\frac{1}{2}\left(a_{0}k_{1}-b_{0}+k_{3}\right)\frac{m_{3}}{m_{2}k_{2}\sqrt{2\pi f_{r}}}$$

$$\eta =\frac{1}{2}\left(b_{0}k_{1}-a_{0}+k_{3}\right)\frac{c_{3}\sqrt{2\pi f_{r}}}{c_{2}k_{2}}$$

with:

$$a_{0}=\frac{1}{m_{3}{\left(2\pi f_{r}\right)}^{3/2}}-\frac{m_{1}}{m_{3}}\sqrt{2\pi f_{r}},\;b_{0}=\frac{1}{c_{3}Q{\left(2\pi f_{r}\right)}^{3/2}}-\frac{c_{1}}{c_{3}\sqrt{2\pi f_{r}}},$$

$$k_{1}=1-2\frac{m_{2}c_{2}}{m_{3}c_{3}},\;k_{2}=1-\frac{m_{2}c_{2}}{m_{3}c_{3}},\;k_{3}=\sqrt{{\left(a_{0}-b_{0}\right)}^{2}+4a_{0}b_{0}\frac{m_{2}c_{2}}{m_{3}c_{3}}}$$

**Figure 3:**
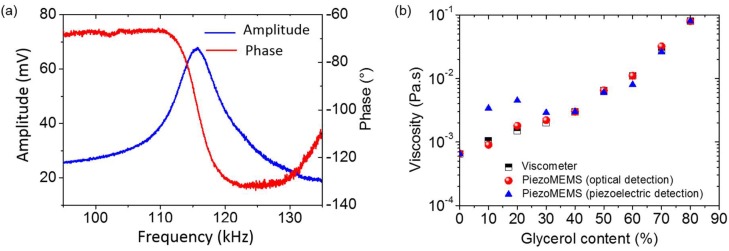
(**a**) Amplitude and phase of the roof-tile-shaped mode resonant frequency; (**b**) Comparison of viscosity values obtained with a commercial viscometer (mass density has to be known for such measurements) and the piezoelectric MEMS using optical and piezoelectric detections (no information on the fluid properties is necessary).

The viscosity of water/glycerol mixtures containing different concentrations of glycerol was determined from the measurement of both the resonant frequency and the quality factor of the piezoelectric polymeric resonators. To do so, a roof-tile-shaped resonance mode was chosen to extract viscosity values (Figure 3a). Nevertheless, a difference between viscosity values measured with a viscometer and the piezoelectric MEMS was observed at low viscosity values (Figure 3b). In fact, optical measurements were obtained for the first flexural mode of the piezoelectric cantilevers, which is the one providing the best analytical comparison with the viscometer. Afterwards, measurements were made with the electronic scheme, but for the roof-tile resonance mode, as it is the one that presents the best electrical response. However, due to electrical coupling that is not entirely compensated, the measurements of the quality factor are misrepresented, which has a direct influence on the extraction of the viscosity values. At low viscosity values, *Q* being higher, even small error in the measurement of *Q* might result in large variation of viscosity measurement of the characterized fluid. Except for small viscosity values (below 5 mPa.s), the values determined with the piezoelectric MEMS using optical and piezoelectric detection showed maximum relative errors of 22% and 32%, respectively, over a large viscosity range. It is noteworthy that despites viscometers requiring the mass density of the fluid to be characterized, piezoelectric MEMS allow unknown fluid viscosity measurements without their properties. These results demonstrated the ability of integrated actuation and read-out of polymeric piezoelectric resonators in liquid media for biological and chemical sensing applications.

### 3.3. Piezotransistive Transducer from P(VDF-TrFE)-Gated OFET

**Figure 4:**
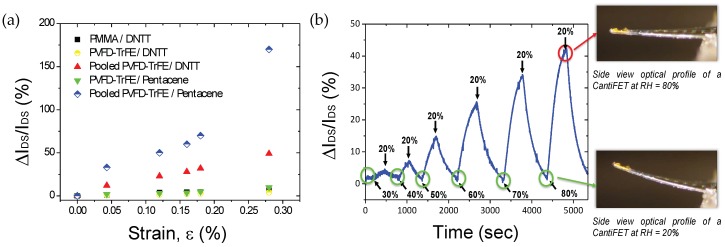
(**a**) Relative changes of drain current (∆*I_DS_*/*I_DS_*) as a function of applied strain for different coupled gate dielectric/OSC layers in OFET-embedded cantilever; (**b**) (∆*I_DS_*/*I_DS_*) plotted versus elapsed time for different levels of relative humidity by steps of 10%. Black arrows correspond to the instruction of humidity level set by the environmental chamber.

While integrated polymer MEMS showed powerful behavior when used in dynamic mode, operating such devices in static mode takes benefits from the large flexibility offered by polymer materials. Integration of highly sensitive transduction schemes in static mode remains a challenge, where simple piezoresistive gauges are currently used, as shown in Section 3.1. To further demonstrate that more complex transduction schemes with enhanced performances can be designed and fabricated, a piezoelectric transistive transduction scheme was developed by integration of a piezoelectric polymer gated organic field effect transistor, OFET, embedded into a polymeric cantilever operating in static mode. The performance of this innovative electromechanical transduction mechanism was evaluated by testing the MEMS as strain sensors. Under applied mechanical loads, the OFET-embedded cantilever bends, resulting in a polarization of the gate induced by the piezoelectric gate dielectric. The piezoelectric effect causes a change of charge density in the semiconductor channel of the OFET, leading to amplified modulation of the drain current, *I_DS_*. In other words, the sensing mechanism of the electro-mechanical transducer originates from the piezoelectric material itself, which affects the electrical behavior of the transistor as signature of a mechanical event. The impact of strain on the characteristics of polarized OFET-embedded cantilevers was investigated and compared to unpolarized OFET to highlight the influence of the piezoelectric gate dielectric layer. Tensile strain cycle measurements were performed by applying and releasing crescent forces to the devices while recording simultaneously drain current variations (*V_DS_*  =  −5 V and *V_GS_ * = −50 V). Steady-state relative drain current variations (Δ*I_DS_*/*I_DS_*) of devices that were not polarized were small compared to those polarized, namely, 9% and 170%, respectively, for an identical applied strain value of 0.28% (Figure 4a). Their corresponding electro-mechanical sensitivities, defined as ((Δ*I_DS_*/*I_DS_)*/*ε*), were calculated to be 33 and over 600. The large enhancement of the strain sensitivity close to the threshold voltage, *V_th_*_,_ by a factor 18 after poling clearly demonstrated the benefit offered by the piezoelectric effect. It is the change of polarization of P(VDF-TrFE) that affects the charge density in the pentacene layer.

To ensure that the enhancement of the electromechanical sensitivity originates from the piezoelectric gate dielectric material, various OFET embedded MEMS structures were fabricated (Figure 4a). First, control devices made with PMMA as control passive dielectric were fabricated. Also, different organic semiconductors (OSC) small molecules were tested (pentacene and DNTT). Prior polarization, P(VDF-TrFE) is not piezoelectric, and therefore behaves like a passive gate dielectric such as PMMA. This behavior was confirmed by the determination of the homogeneity of the sensitivity of devices made of PMMA/DNTT, unpoled P(VDF-TrFE)/DNTT and unpoled P(VDF-TrFE)/pentacene as 25, 14 and 33, respectively. However, once the P(VDF-TrFE) layer was polarized, a drastic increase of the strain sensitivity by a factor 18, regardless of the OSC materials, was seen. Among polarized devices, pentacene-based OFETs presented the largest sensitivities. It is well-known that strain-sensitivity of OSC is highly dependent on its morphological structure. Thus, the record sensitivities observed in the piezoelectric OFET-embedded MEMS was due to a combination of piezoelectricity induced in the active gate dielectric and a strain-dependent mobility of the OSC.

To further demonstrate potential applications of the piezoelectric OFET transducer, the devices were tested as humidity sensors. For this, the cantilevers were coated with a hydrogel thin film employed as the sensitive layer. The hydrogel was synthesized by free radical polymerization of hydroxyethyl-methacrylate (HEMA) and ethylene-glycol-dimethacrylate (EGDMA) monomers where EGDMA acts as a cross-linker. The large volume change due to water molecule absorption leads to surface strain experienced by the cantilever due to bi-layer effect that in turn, results in large *I_DS_* modulations proportional to the relative level of humidity (Figure 4b). The sensitivity of this sensor was measured to be 7500 ppm/%RH, with an extracted limit of detection of 0.2%RH. These results highlight the benefits offered by the MEMS configuration where the piezoelectric OFET amplifies the sensor’s response. They confirmed the ability of the piezoelectric OFET-embedded MEMS to monitor steady state sensing events, and thus show promise for future monitoring of complex sensing events, such as biological analysis.

## 4. Conclusions

Polymers and their composites represent one of the most important functional engineering materials, and are used in an increased number of applications, now, including microsystems. This work highlights the recent advancements of our team in the field of polymer materials and technology for their integration as electromechanical transduction schemes in polymer MEMS and compares their main characteristics. Table 1 depicts the main characteristics of the presented electromechanical transduction methods. The electrical output characteristics can be passive, such as piezoresistive, or active, i.e., piezoelectric and piezotransistive transducers. Passive resistive measurements require a Wheatstone bridge electronics configuration, and thus are generally more complex to integrate into polymer MEMS. On the other hand, the ease of measurement of voltage or current variations offered by piezoelectric and piezotransistive transductions stand as an undeniable advantage in terms of implementation. Nevertheless, the relatively low sensitivity of piezoelectric polymers (d_31_ = 10 to 12 pm·V^−1^) and the influence of parasitic capacitive effects require conditioning electronic such as charge amplifier and coupling effects attenuation to allow accurate dynamic measurements. Definitely, one of the main pressing concern for polymer MEMS sensors is their sensitivity. Piezoresistive polymeric MEMS with high gauge factor values (~200) were reported by taking advantage of the composite approach. However, these piezoresistive nanocomposite-based sensors might suffer from relatively low stability and reproducibility due to the large discrepancy of their sensitivity in the percolation region, which is generally the concentration of interest in achieving high gauge factor. In the case of piezoelectric polymers, the commercially available P(VDF-TrFE) stands as the material of choice, and presents the advantage of exhibiting more reproducible characteristics. One of the main advantages of using piezoelectric materials lies in the reversible piezoelectric effect, which allows integrated actuation and read-out for dynamic mode measurement. To overcome the limited sensitivity of piezoelectric polymers, we developed an original approach that consisted of the integration of piezoelectric P(VDF-TrFE) as a gate dielectric layer in an organic field effect transistor (OFET) to take advantage of the amplification of the transistor. The obtained piezotransistive MEMS strain sensor exhibited a sensitivity of 600. The presented piezoresistive, piezoelectric and piezotransistive electromechanical transduction mechanisms and their integration into multilayer polymeric cantilevers demonstrate the potential of polymer integrated electromechanical transduction schemes to fulfill the needs of a variety of physical and chemical sensors. This work should encourage further research on the development of polymeric MEMS and their use in other fields of application, e.g., biological sensors, actuators and energy harvesting.

## Figures and Tables

**Table 1:** Main characteristics of piezoresistive, piezoelectric and piezotransistive transductions.

MEMS.	Transduction	Actuation	Micromachining	Operating Mode	Sensitivity Max	Applications
Piezoresistive cantilever [5,17,18,19,20,21,22,23,24,25,26,27,28,29]	Piezoresistive	-	Photo-patternable Bulk stacking	Static and Dynamic	200 [28]	Strain, temperature, gas sensing
Piezoelectric resonator [31,32,33,34,35,36,37]	Piezoelectric	Piezoelectric	Bulk stacking	Dynamic	NA	Liquid sensing, energy harvesting
OFET-embedded cantilever [45,46]	Piezotransistive	-	Bulk stacking	Static	600 [46]	Strain, gas sensing
